# Recognizing and managing the expanded risk of tumor lysis syndrome in hematologic and solid malignancies

**DOI:** 10.1186/1756-8722-5-75

**Published:** 2012-12-13

**Authors:** Ali McBride, Peter Westervelt

**Affiliations:** 1 Arthur G. James Cancer Hospital, The Ohio State University, Department of Pharmacy, Room 368 Doan Hall, Columbus, OH, 43210, USA; 2 Department of Medicine, Oncology Division, Washington University, St. Louis, MO, USA

**Keywords:** Acute renal failure, Allopurinol, Adverse events, Hematologic malignancies, Management, Prophylactic therapy, Solid tumors, Tumor lysis syndrome (TLS), Rasburicase, Uric acid

## Abstract

Tumor lysis syndrome (TLS) is widely recognized as a serious adverse event associated with the cytotoxic therapies primarily used in hematologic cancers, such as Burkitt lymphoma and acute lymphoblastic leukemia. In recent years, TLS has been more widely observed, due at least in part to the availability of more effective cancer treatments. Moreover, TLS is seen with greater frequency in solid tumors, and particularly in bulky tumors with extensive metastases and tumors with organ or bone marrow involvement. The consequences of TLS include the serious morbidity and high risk of mortality associated with the condition itself. Additionally, TLS may delay or force an alteration in the patient’s chemotherapy regimen. The changing patterns of TLS, as well as its frequency, in the clinical setting, result in unnecessarily high rates of illness and/or fatality. Prophylactic measures are widely available for patients at risk of TLS, and are considered highly effective. The present article discusses the various manifestations of TLS, its risk factors and management options to prevent TLS from occurring.

## Introduction

In recent years, tumor lysis syndrome (TLS), an oncologic emergency typically associated with cytotoxic therapies, is more likely to be seen across a spectrum of cancer types [1-3]. Previously regarded as a risk primarily in hematologic malignancies such as Burkitt lymphoma and acute lymphoblastic leukemia (ALL), TLS is now observed in malignancies that had rarely been associated with TLS, including solid tumors [4-9]. This change in pattern is likely the result of several factors including the availability of effective cytotoxic therapies for a wider range of malignancies, as well as an insufficient use of prophylactic therapies to adequately prevent TLS [1]. Although healthcare providers have expressed concerns regarding the TLS risk related to newer chemo modalities, they are not consistently utilizing straightforward measures for reducing TLS risk in their extended spectrum of patients at risk for TLS [2,10,11]. With increasingly powerful chemotherapy agents being used to treat patients, it is more important than ever that patients undergo risk assessment for TLS in order that they may receive appropriate treatment to reduce the risk of occurrence. In the present article, we explore several key areas relevant to the evolving knowledge of TLS prevention that reflect the changing nature of the disease in the current clinical setting, and some frequently overlooked issues important to an understanding of TLS. In addition, we review the current and changing approaches to risk assessment and management of TLS.

### Definition of TLS

TLS occurs when the cellular components of tumor cells are released into the blood after lysis, typically after chemotherapy or radiation therapy [10]. It is characterized by hyperuricemia, hyperkalemia, hyperphosphatemia, and hypocalcemia, factors which may overtax the body’s homeostatic mechanisms and overwhelm the capacity for normal excretion of these materials [10,12]. This, in turn, causes various manifestations of TLS, including acute renal failure [10,12] and cardiac arrest due to electrolyte abnormalities [10]. Malignancies, which typically result in TLS, are ones that possess a high proliferation rate and/or a large tumor burden, such as lymphomas and acute leukemias [10,13]. Moreover, patients whose melanoma is particularly sensitive to chemotherapy are also more likely to experience TLS [13]. Spontaneous TLS – that is, TLS occurring in the absence of cytotoxic therapy – is another concern among patients with malignancies who are at risk for TLS, and many of the same risk factors and preventive measures appropriate for TLS also apply to spontaneous TLS [6].

The standard definition for TLS comprises two separate definitions— clinical TLS (CTLS) and laboratory TLS (LTLS) — standardized by Cairo & Bishop in 2004, and based on an earlier definition by Hande & Garrow in 1993 [14,15] (Table 1). In 2011, Howard et al. suggested revisions to the Cairo & Bishop definitions [1]. The modified Howard definition of LTLS is ≥2 of the following metabolic abnormalities occurring simultaneously within 3 days prior to and up to 7 days after treatment initiation: hyperuricemia (>8.0 mg/dl), hyperkalemia (>6.0 mmol/liter), hyperphosphatemia (>4.5 mg/dl), and hypocalcemia (corrected Ca <7.0 mg/dl, ionized Ca <1.12 mg/dl). The modified Howard definition for CTLS is the same as laboratory-defined TLS, and is accompanied by elevated creatinine level, seizures, cardiac dysrhythmia, or death. In addition, any symptomatic hypocalcemia is considered diagnostic [1].

**Table 1:** Comparison of tumor lysis syndrome (TLS) definitions

**Reference**	**Laboratory TLS**	**Clinical TLS**	**Other**
**Hande & Garrow 1993**[15]**]**	≥2 of the following metabolic abnormalities occurring within 4 days of treatment:	Laboratory-defined TLS accompanied by any of the following:	_
	○ 25% increase from baseline in UA	○ Creatinine level >221 μmol/l (2.5 mg/dL)	
	○ 25% increase from baseline in potassium	○ Potassium level >6 mmol/L (6 mEq/L)	
	○ 25% increase from baseline in phosphate	○ Calcium <1.5 mmol/L (6 mg/dL)	
	○ 25% decline from baseline in calcium	○ Development of a life-threatening arrhythmia	
		○ Sudden death	
**Cairo & Bishop 2004[**[13]**]**	≥2 of the following metabolic abnormalities occurring simultaneously within 3 days prior to and up to 7 days post-treatment initiation:	Laboratory-defined TLS accompanied by any of the following:	_
	○ UA ≥476 μmol/L or 25% increase from baseline	○ Elevated creatinine level (≥1.5 ULN for patients >12 years of age or age-adjusted)	
	○ Potassium ≥6.0 mmol/L or 25% increase from baseline	○ Seizures	
	○ Phosphorous ≥2.1 mmol/L (children) ≥1.45 mmol/L (adults) or 25% increase from baseline	○ Cardiac dysrhythmia	
	○ Calcium ≤1.75 mmol/L or 25% decrease from baseline	○ Death	
**Howard SC, et al. 2011[**[1]**]**	≥2 of the following metabolic abnormalities occurring simultaneously within 3 days prior to and up to 7 post-treatment initiation:	Laboratory-defined TLS accompanied by any of the following:	Any symptomatic hypocalcemia is diagnostic
	○ UA >8.0 mg/dL (475.8 μmol/L) or above ULN for age in children	○ Elevated creatinine	
		○ level	
	○ Potassium >6.0 mmol/L	○ Seizures	
	○ Phosphorus >4.5 mg/dL (1.5 mmol/L) or >6.5 mg/dL (2.1 mmol/L) in children	○ Cardiac dysrhythmia	
		○ Death	
	○ Corrected* calcium <7.0 mg/dL (1.75 mmol/L) or ionized calcium <1.12 mg/dL (0.3 mmol/L)		

UA, uric acid; ULN, upper limit of normal.

*Corrected calcium in mg/dL = measured calcium level in mg/dL + 0.8 × (4 – albumin in g/dL).

### TLS in solid tumors

Although TLS has long been assumed to manifest primarily in hematologic malignancies, case reports of TLS in solid tumors have become increasingly common over the last decade [4-9]. The diversity of these reports is too broad to report comprehensively; however, below are several examples of the occurrence of TLS in varying types of solid tumors.

A 2006 publication by Mott et al. reported LTLS in three different patients—two with breast cancer and one with small cell carcinoma [16]. A 47-year old woman with metastatic breast cancer previously treated with doxorubicin and docetaxel developed TLS with diagnosis based on increased uric acid (UA) and lactate dehydrogenase (LDH), after initiating treatment with fluorouracil (5FU), epirubicin and cyclophosphamide (FEC). Her LDH and UA—though unrecorded prior to chemotherapy—reached 916 IU/dL (normal range 60–200) and 10 mg/dL (normal range 2.4-7.9), respectively, after one day of treatment. These levels decreased to some extent by the evening of day 2, and although the UA level normalized, LDH remained well above the normal range [16]. In the second case, a 44-year old woman with breast cancer initially treated with docetaxel without complication developed TLS, after gemcitabine plus cisplatin was initiated for metastatic disease. The patient’s laboratory values were significant for elevated LDH, phosphorus, potassium, UA, creatinine, and decreased calcium after 4 days of carboplatin and etoposide. Also reported was a 76-year-old woman with small cell carcinoma who developed elevated UA, serum potassium, phosphorus, and decreased calcium after 4 days of carboplatin and etoposide [16].

TLS in non-small cell lung cancer (NSCLC) — both squamous cell carcinoma and adenocarcinoma — has been reported in several instances, including after treatment with docetaxel, zoledronic acid, radiotherapy, and in at least one case, arising spontaneously [6-9]. A patient with metastatic colon cancer, for whom chemotherapy had been ruled out due to liver metastases causing hyperbilirubinemia and transaminitis, underwent treatment with the monoclonal antibody cetuximab [17]. Renal function deteriorated after 18 hours, and the patient experienced elevations in UA, phosphorus, potassium, and decreased calcium, consistent with CTLS.

Intense tumor lysis (though not diagnostic for TLS) was seen in a 33-year-old patient with hepatocellular carcinoma who was treated with sorafenib, a tyrosine kinase inhibitor [4]. Four days after treatment initiation, he experienced fatigue and fever; laboratory studies found that compared with pre-treatment baseline, his potassium had increased and calcium decreased, although creatinine and phosphorus were roughly unchanged and his UA had decreased [4].

A 44-year-old patient with primary retroperitoneal soft-tissue sarcoma was given a combination chemotherapy regimen of cisplatin, adriamycin, and dacarbazine after a chemosensitivity assay revealed that the malignancy was sensitive to these agents [5]. After 4 days, the patient experienced palpitations, dyspnea, chest tightness, and oliguria, accompanied by abnormally high creatinine as well as hyperuricemia, hyperphosphatemia, hypocalcemia diagnostic for CTLS, and acute renal failure. CTLS was also reported in a 60-year-old patient with recurrent endometrial cancer who had been receiving carboplatin and paclitaxel [18]. Four days after receiving treatment with both agents, she presented to the emergency room with dyspnea, weakness, fatigue, metabolic and electrolyte abnormalities, as well as UA, potassium, and phosphate levels consistent with TLS [18].

Case reports of TLS resulting from the treatment of metastatic melanoma (MM) have also been published. A 56-year-old patient with abdominal pain, vomiting, and weight loss, ongoing for 2 months, was diagnosed with MM and treated with intravenous hydrocortisone for hypercalcemia [19]. By the following day, lab values indicated the onset of CTLS; treatment for TLS along with discontinuation of the hydrocortisone resulted in a resolution of symptoms [19]. A 61-year-old patient presented with a nevus in the abdominal wall, and melanoma extending to lateral margin and invading the lymphatic channels was diagnosed and excision performed [20]. Six months later, melanoma was found in 6 of 15 lymph nodes, the MM having expanded despite treatment with one cycle of granulocyte macrophage–colony stimulating factor. Chemotherapy of cisplatin, vinblastine, and dacarbazine was initiated, along with interleukin-2 and interferon-α. LDH rose dramatically within 24 hours. By day 3, CTLS symptoms had emerged and the following day chemotherapy was discontinued.

A 41-year-old patient with metastatic melanoma initiated on cisplatin, dacarbazine and interferon developed oliguria on day 2 after chemotherapy and symptoms of CTLS by day 4; acute renal failure developed shortly thereafter [21]. The potential greater potency of new chemotherapeutic regimens may be associated with a heightened risk for TLS.

### Spontaneous TLS

The term “spontaneous TLS” refers to manifestations of TLS in patients who have not received cytotoxic therapy [6]. As with treatment-related TLS, spontaneous TLS was thought to be primarily confined to hematologic cancers [6]. While this is largely the case, incidences of spontaneous TLS in solid tumors have been reported [11,13]. Case reports of spontaneous TLS in hematologic cancers include cases occurring in patients with Burkitt lymphoma, non-Hodgkin’s lymphoma, acute myeloid leukemia, B-cell lymphoma, and ALL, among others [11,22-27]. Solid tumors in which spontaneous TLS has been observed include breast cancer, gastric cancer, germ cell tumors, gastrointestinal adenocarcinoma, squamous cell lung carcinoma, and metastatic castrate-resistant prostate cancer [6,28-32]. Hyperphosphatemia is less common in spontaneous than nonspontaneous TLS, possibly because phosphate release in lysis is less achievable when cytotoxic therapy has taken place [33].

### TLS risk factors

**Figure 1:**
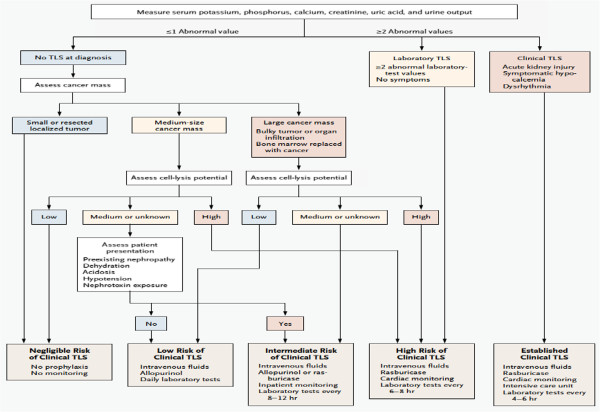
**Tumor lysis syndrome treatment (TLS) stratification algorithm[**[1]**]** .

**Figure 2:**
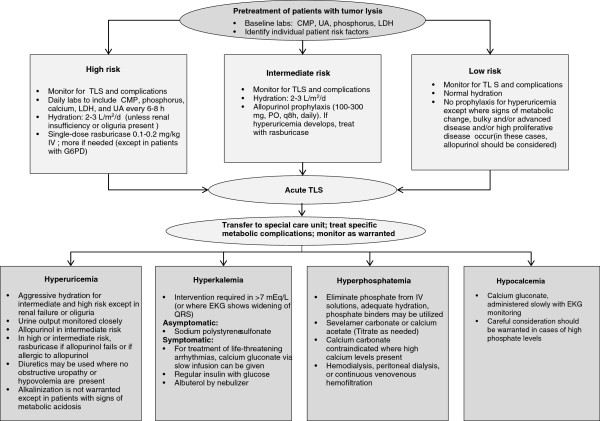
**Algorithm for the management of tumor lysis syndrome (TLS) [**[3]**,**[13]**,**[34]**].** CMP, complete metabolic panel, EKG, electrocardiogram; G6PD, glucose-6-phosphate dehydrogenase; IV, intravenous; LDH, lactic dehydrogenase; PO, by mouth.

Risk assessment is fundamental to the management of TLS, particularly in light of the highly effective prevention and treatment options available to clinicians. While general risk factors for TLS are typically well understood, stratifying patients with specific malignancy types, at specific disease stages, with particular disease manifestations is a considerably more complex task [13]. Cairo et al. developed several risk assessment models that allow for risk estimation based on cancer type as well as several key factors, including choice of chemotherapy, state of renal function, and disease stage, among other considerations [34]. These models, while informative, may be challenging to implement in the clinical setting, in part because they comprise 6 separate algorithms. Howard et al. developed a single simplified algorithm for risk assessment together with recommended therapy which, while less detailed, is somewhat more accessible for clinical purposes [1] (Figure 1). An adaptation of an algorithm developed by Wetzstein, for an overall approach to the management of TLS, is seen in Figure 2.

Risk factors for TLS related to tumor size and expansion include bulky tumor, wide metastatic dispersal, and organ and/or bone marrow involvement [1,13]. TLS risk is increased when a high potential for cell lysis exists; for example, in cases of high proliferation and tumor sensitivity to particular cytotoxic therapies, and during times when therapy intensity is particularly high [35]. Patients’ health status, beyond malignancy-related factors, can also influence the risk of TLS, including presence of hypotension, dehydration, acidic urine (because of the greater propensity of UA to crystalize at low pH), oliguria, pre-cancer nephropathy, and previous experience with nephrotoxic agents [1,36]. Medications and other compounds that tend to increase UA levels (Table 2) are additional risk factors for TLS.

**Table 2:** **Compounds associated with increasing uric acid in the body**[37,38]

Alcohol	Diazoxide	Methyldopa
Ascorbic acid	Diuretics (Thiazide)	Nicotinic acid
Aspirin	Epinephrine	Pyrazinamide
Caffeine	Ethambutol	Phenothiazines
Cisplatin	Levodopa	Theophylline

### Considerations in the management of TLS

Several key considerations and specific tasks are fundamental in the management of TLS. These include risk assessment, fluid management for TLS prophylaxis, and appropriate drug therapy for prophylaxis and TLS treatment. In addition, where rasburicase therapy will be applied, ongoing debates and current knowledge regarding appropriate dosage amounts and approaches to dosing (eg, flat dosing versus weight-based dosing) must be taken into consideration.

Clinical experience suggests that provision of appropriate prophylactic therapy for TLS may be the difference between successful and unsuccessful outcomes in at-risk patients [1]. Appropriate management of TLS should be centered around risk assessment of cancer patients, preventive treatment where appropriate, electrolyte monitoring in patients undergoing cytotoxic therapy, and rapid appropriate therapeutic intervention as necessary [10].

Fluid management is key in the prevention of TLS [13]. This involves both the vigorous application of hydration and diuresis to maintain a flow of urine that will dispose of systemic UA and phosphate. Urine alkalinization with sodium bicarbonate had been a standard approach in TLS management to increase urate excretion [14]. Alkalinization is, however, associated with a reduction in the solubility of calcium phosphate, thus potentially creating the problem in the setting of hyperphosphatemia, a more serious condition than the one it aims to treat [1,13]. The 2008 guidelines for the management of TLS state that sodium bicarbonate is no longer recommended for TLS management [13]. The rationale for this recommendation is that although alkalinization promotes UA excretion, it has a relatively small impact on xanthine and hypoxanthine solubility. Allopurinol, a cornerstone of TLS prevention, is used to prevent formation of UA. It decreases the formation of UA by inhibition the enzyme (XO) that converts xanthine to hypoxanthine to UA. Inhibition of XO leads to increased levels of xanthine and hypoxanthine. Therefore, due to the risk of both xanthine crystallization, calcium phosphate precipitation, as well as the occurrence of metabolic alkalosis associated with alkalization, the utility of routine use of sodium bicarbonate for the prevention of TLS has fallen out of favor [13]. Taken together, this risk plus the risk of calcium phosphate precipitation, as well as that of the metabolic alkalosis associated with alkalinization, challenges the clinical utility of sodium bicarbonate. It is also the case that in patients being treated with rasburicase, alkalinization has been associated with the potential risk of acute renal failure, and the 2008 TLS guidelines regard alkalinization as contraindicated in patients treated with rasburicase [13,39].

Allopurinol is commonly used in TLS management to reduce the conversion of xanthine and hypoxanthine to UA, a process for which it is highly effective [13]. Allopurinol is, however, ineffective at reducing UA formed prior to treatment, and its slow time to efficacy can necessitate delaying chemotherapy or reducing the dose of chemotherapy for patients in acute renal failure. Due to its low levels of solubility, allopurinol, by increasing systemic levels of xanthine and hypoxanthine, can also promote obstructive uropathy [40]. Reduced clearance of purine-based chemotherapeutic drugs is an additional feature of allopurinol that may require the dose reduction of these chemotherapeutic agents [1,13].

Rasburicase, the first recombinant uricolytic agent, rapidly reduces UA levels by eliminating existing UA [1,41]. The efficacy of rasburicase in depleting UA involves its enzymatic degradation of UA into allantoin, which is highly soluble and is not associated with adverse effects in human patients [1]. Investigators have demonstrated rasburicase to be safe and effective for prophylaxis or treatment of hyperuricemia in patients with leukemia or lymphoma [41,42]. Rasburicase is FDA approved for initial management of pediatric and adult patients with leukemia, lymphoma, and solid tumor malignancies who are receiving anticancer therapy expected to result in tumor lysis and subsequent elevation of plasma UA [43]. Rasburicase is recommended as a first-line therapy for patients at high risk of TLS, and is also used in Europe to treat intermediate-risk adult patients [13].

It should be noted that rasburicase is contraindicated in patients with a glucose-6-phosphate dehydrogenase (G6PD) deficiency as these patients are at an elevated risk for hemolysis [43]. Patients who are more likely to have a G6PD deficiency include African Americans and some people of Mediterranean and Southeast Asian descent [13].

The potential benefits of using rasburicase in sequential combination with allopurinol was explored in an open-label phase III study in which 275 patients with hematologic malignancies were randomized to receive allopurinol (300 mg/d) or rasburicase (0.20 mg/kg/d) or both over a period of 5 days [2]. The sequential combination group received rasburicase on days 1 through 3 and allopurinol on days 3 through 5 with an overlap on day 3. The response rates with regard to serum UA were 87% for those treated only with rasburicase, 78% for those treated with the combination, and 66% for allopurinol monotherapy [2]. Rasburicase was significantly more effective than allopurinol (*P*=0.001), while the combination did not reach statistically significant superiority over allopurinol alone (*P*=0.06). Similar results were observed in subgroups of patients at elevated risk for TLS and for those with hyperuricemia at baseline. Treatment-related AEs were rare and similar between treatment groups. Two subjects in each of the monotherapy groups experienced acute renal failure (2% for each group), while 5 subjects (5%) in the combination therapy group experienced acute renal failure [2].

### Dosing of rasburicase

The ideal method of dosing rasburicase has been an area of some debate, with one-time dosing, either as a fixed or weight-based dose, being preferred by many over weight-based, multi-dose therapy. Indeed, despite the FDA’s dosing recommendation of 0.2 mg/kg/d for up to 5 days, most rasburicase prophylactic treatment in the United States employs a flat dose of 3 mg to 7.5 mg daily [43]. A series of small studies have demonstrated the efficacy of a single fixed or weight-based dose of rasburicase in reducing UA in TLS patients or patients at high risk for TLS. Fixed-doses employed in these studies were 3 mg, 6 mg, and 7.5 mg. Weight-based dosing was either 0.15 or 0.05 mg/kg [44-51]. A retrospective review from 2006 examined the efficacy of a fixed 3 mg dose of rasburicase given to 43 patients with hematologic malignancies who were receiving chemotherapy or hematopoietic stem cell transplantation. All subjects in the study were hyperuricemic, with 15 patients having laboratory values suggestive of TLS and the remainder at elevated risk for TLS. Patients were given allopurinol “as required”, to suppress UA formation. Most patients experienced a significant decline in UA within the first 24 hours, and 6 subjects required an additional dose of rasburicase: 2 received a 1.5 mg second dose and 4 received a 3 mg second dose. Within 48 hours, UA had normalized in all patients and none required a third dose [51].

A retrospective review from 2009 assessed the use of a weight-based approach to rasburicase therapy in 21 cancer patients, with dosing based on ideal body weight (n=11); in cases where a patient was in excess of 30% of the IBW (n=10), an adjusted dose was given. The average initial dose administered was 0.15 mg/kg ± 0.03. All patients in the study had laboratory values reflecting TLS or high risk for TLS, and all patients received allopurinol. Within 6 hours of treatment, the mean reduction from baseline of UA was 65.3% ± 17.3, and within 24 hours UA levels had been reduced by 89.7% ± 9.0%. No data regarding additional doses was reported [47]. Fixed-dose efficacy has also been shown in a small number of patients with spontaneous TLS [45].

A recently published chart review from our institution of single fixed-dose and weight-based dosing of rasburicase in 373 evaluated patients with malignancies, but at varying levels of risk for TLS, sought to determine the efficacy of these approaches to dosing in a larger and more diverse patient population [52]. The primary endpoint of this chart review was normalization of UA at 24 hours; secondary endpoints were UA normalization at 48 and 72 hours [52]. Treatment across all groups was found to be highly effective, with only 6 study subjects failing to achieve normalized UA levels within 24 hours. There were no significant differences between dosing groups for any of the endpoints, although 3 mg was found to have a weaker effect on UA reduction. That is, while the 3 mg dose was equally effective at achieving treatment success (i.e., <7.5 mg/dL within 24 hours), the mean UA level at 24 hours in the 3 mg group was 3.69 mg/dL compared to 1.71 mg/dL , 1.42 mg/dL , and 1.03 mg/dL in the 6 mg, 7.5 mg, and weight-based dosing groups, respectively [52]. No significant differences between low, intermediate, and high-risk patient groups were observed at 24 or 72 hours, while such a difference was observed at 48 hours in the low-risk group (P=0.017) [52].

A recent randomized, open-label clinical trial compared two rasburicase regimens in 80 patients at high risk for TLS (defined as presence of hyperuricemia or very aggressive lymphoma or leukemia) or potential risk (defined as aggressive lymphoma or leukemia plus LDH ≥upper normal limit, or stage or stage ≥3 disease, or stage 1 or 2 disease with ≥1 lymph node/tumor >5 cm) [53]. The regimens were 0.15 mg/kg given as a single dose followed by as-needed dosing versus the same dose given daily for 5 days. All but 1 patient experienced normalized UA within 24 hours, and UA reached undetectable levels within 4 hours for 84% of the study subjects. UA levels were largely sustained in both groups with the notable exception of 5 patients in the high-risk, single-dose arm who required a second dose during the 5-day study period. Two of these patients required a second dose on day 3, 1 patient on day 4, and 1 patient on day 5. All 5 of these patients had very aggressive lymphoma and/or bulky tumor, including 3 with diffuse large B-cell lymphoma, 1 with Burkitt lymphoma, and 1 with Burkitt-like disease. No patients required a third dose [53].

Experience with rasburicase has shown it to be largely well tolerated, with side effects of this agent tending to cluster around hypersensitivity/allergic reactions. These include rash/pruritus, methemoglobinemia, fever, neutropenia, hypoxia, and, rarely, anaphylactic shock. Anemia can also occur, and, as previously noted, patients with G6PD deficiency should not be treated with rasburicase [52-55]. In the one head-to-head open-label study in which treatment with a single dose of rasburicase was compared to five daily doses, the incidence of the most common side effects—generally mild to moderate in severity (eg, nausea, constipation, diarrhea, and vomiting)—was notably less in the single-dose treatment group [53].

## Conclusions

The development of a wider range of cytotoxic therapies and the application of, in some cases, more intensive therapy for the treatment of a variety of cancer types has increased the risk of TLS and the spectrum of its manifestations. Whereas TLS has been widely regarded as associated with hematologic malignancies, an increasing number of reports show that TLS also occurs in solid tumors. In addition, spontaneous TLS is seen more frequently, albeit it represents a minority of TLS cases.

The impact of TLS, as an oncologic emergency, often extends beyond the immediate consequences of the condition itself. In many cases TLS can cause delay of necessary chemotherapy, force a reduction in chemotherapy dosing and alter the selection of cytotoxic agents due to treatment toxicities overlapping with damage induced by TLS. Kidney damage, heart failure, fluid retention, neuromuscular effects, as well as gastrointestinal effects, are examples of damage that can occur. As many practitioners in the clinical setting have yet to fully realize the consequences of TLS, often associated with more effective cancer treatments and newer regimens, the likelihood of cancer patients at risk for TLS is probably underestimated, resulting in unnecessary patient morbidity and mortality [54,55].

With the availability of highly effective treatments for the prevention and management of TLS, it is worth reiterating the importance of risk assessment in cancer patients. Clinical experience has definitively demonstrated that the provision of appropriate prophylactic therapy for TLS at the appropriate dose can be the difference between successful and unsuccessful outcomes in at-risk patients. Preventive and therapeutic interventions for TLS have been shown to be highly effective and relatively easy to implement. (See Table 3 for a synopsis of pharmacologic therapies for the treatment of TLS).

**Table 3:** **Pharmacologic therapies for the treatment of tumor lysis syndrome (TLS)**[3]

	**Medication**	**Mechanism of Action**	**Dosage/Administration**	**Comments**
**Hyperuricemia**	**Allopurinol**	Potent inhibitor of xanthine oxidase, the enzyme responsible for the conversion of hypoxanthine to xanthine to uric acid.	PO: 200–300 mg/m^2^ qd; administration of >300 mg should be given in divided doses (max, 800 mg/d); should initiate therapy 24 to 48 hours prior to chemotherapy.	Adverse events include maculopapular rash, dyspepsia, nausea/ vomiting, fever, and eosinophilia; rare reports of interstitial nephritis; decreases in serum uric acid occur in 1 to 2 days with a nadir ~7 days; dosage adjustment in renal dysfunction is necessary to avoid accumulation of the active metabolite oxypurinol (alloxanthine); removed by dialysis, so administer post-hemodialysis or administer 50% supplemental dose; significant drug interactions with azathioprine and 6-mercaptopurine; the dose of concomitant azathioprine or 6-mercaptopurine should be reduced to one third to one fourth of their usual dose.
			Adult (IV): 200–400 mg/m^2^/d as a single infusion or divided doses (max, 600 mg/d); infuse over 15–60 minutes; final concentration no greater than 6 mg/mL.	
			Pediatric (IV): Starting dose 200 mg/m^2^/d.	
	**Rasburicase**	Recombinant protein that catalyzes enzymatic oxidation of uric acid into an inactive metabolite, allantoin, that is 5 to 10 times more soluble than uric acid.	Adult: 0.2 mg/kg infusion over 30 minutes once daily for up to 5 days*; no dosing adjustment required in renal or hepatic dysfunction.	Adverse events include nausea/vomiting, fever, headache, abdominal pain, constipation, diarrhea, and rash. Rare (<1%) but serious reactions have occurred such as severe hypersensitivity reactions, including anaphylaxis, hemolysis, and methemoglobinemia. Caution is advised in patients who have atopic allergies/asthma.
			Pediatric: 0.15-0.2 mg/kg IV infusion over 30 minutes daily × 5 days*; no dosing adjustment required in renal or hepatic dysfunction.	
			*Studies using single dose in the treatment of hyperuricemia has been reported [55].	
				Contraindicated in individuals deficient in glucose-6-phosphatase dehydrogenase (G6PD). Rasburicase will cause enzymatic degradation of uric acid within blood samples left at room temperature, resulting in spuriously low uric acid levels—blood must be collected into prechilled tubes containing heparin anticoagulant and immediately immersed and maintained in an ice water bath; plasma samples must be assayed within 4 hours of sample collection.
**Hyperkalemia**	**Sodium polystyrene sulfonate**	Removes potassium (K+) by exchanging sodium ions (Na+) for K+ in the intestine.	Adult: 15 g PO (60 mL) 1 to 4 times per day	1 g resin binds approximately 1 mEq of K+; onset is variable ~2 to 24 hours; administer orally or nasogastrically with a laxative such as sorbitol to avoid fecal impaction and facilitate elimination; chilling the solution will increase palatability; enema route is usually less effective.
			Pediatric: 1 g/kg/dose PO q6h or q2-6h rectally.	
	**Calcium gluconate**	Raises threshold potential and reestablishes cardiac excitability.	Adult: 1–3 g over 3 to 5 minutes IV push.	Antagonizes the action of hyperkalemia on the heart; should be monitored closely by ECG when given; onset ~1 to 2 minutes; duration is ~10 to 30 minutes.
			Pediatric: 60–200 mg/kg over 3 to 5 minutes slow IV push.	
	**Loop diuretics**	Inhibits reabsorption of Na+ and chloride, thus causing increased excretion of fluid, K+, and phosphate.	Adult: **bumetanide**: 0.5-1 mg IV or PO 1 to 2 times per day (1 mg = 40 mg furosemide); **ethacrynic acid** (PO) or **ethacrynate sodium** (IV [Edecrin, Merck]): 0.5-1 mg/kg/dose IV or PO q8-12h prn; **furosemide**: 20–80 mg/dose IV or PO q6-12h prn; **torsemide**: 10–20 mg IV or PO every day prn (20–30 mg = 40 mg furosemide).	Onset: IV ~5 minutes; PO ~30 to 60 minutes; duration ~6 to 12 hours, depending on agent; monitor for blood pressure, electrolytes, and renal function.
			Pediatric: **bumetanide** (>6 months): 0.015-0.1 mg/kg/dose once or twice daily (safety and efficacy have not been established in children <18); **ethacrynic acid:** 25 mg PO daily (maximum dose, 2–3 mg/kg/d); **furosemide** 1**–**2 mg/kg/dose IV or PO q6-8h prn (maximum daily dose, 40 mg).	
	**Dextrose and regular insulin**	Shifts K+ intracellularly.	Adult: D_5_W at 0.5-1 mL/kg and regular insulin 1 unit for every 4–5 g of dextrose given.	Onset is usually within 30 to 60 minutes; effects are temporary, usually lasting 2 to 6 hours.
			Pediatric: The dosage of regular insulin is 1 unit for every 4–5 g of dextrose; usually dextrose 25% or 50% (0.5-1 g/kg) is used with insulin; dextrose 0.5-1 g/kg can also be infused over 15 to 30 minutes followed by insulin 0.1 unit/kg.	
	**Sodium bicarbonate**	Increases serum pH and causes a temporary shift of K+ into cells.	Adult: 50 mEq as IV bolus, or 50–150 mEq added to 1 liter D_5_W and administered as an infusion.	Indicated for patients with acidosis; onset ~30 to 60 minutes and may last 2 to 6 hours, but effects are temporary (Sodium acetate may be substituted for Sodium bicarbonate when in shortage).
			Pediatric: When sodium bicarbonate is determined to be necessary, an initial dose of 1 mEq/kg may be given initially either IV or per intraosseous route, followed by not more than half of that dose every 10 minutes as needed.	
**Hyperphosphatemia**	**Calcium acetate (PhosLo, Nabi)**	Binds to phosphate taken in through diet to form insoluble calcium phosphate, which is then excreted from the body without being absorbed.	Adult: 2 tablets or gelcaps (667 mg) with each meal; dosage may be increased gradually to bring serum phosphate value <6 mg/dL as long as hypercalcemia does not develop.	Adverse events include nausea, mild hypercalcemia (manifested as constipation, anorexia, nausea and vomiting), severe hypercalcemia (associated with confusion, delirium, stupor, coma), and pruritus. Not recommended unless patient is symptomatic because of potential for Ca/PO_4_ precipitates to form, especially if alkalinizing the urine.
	**Sevelamer (Renagel, Genzyme)**	Cross-linked poly(allylamine hydrochloride) is a cationic polymer that binds intestinal phosphate. The compound contains multiple amines that are protonated in the intestinal tract and interact with phosphate via ion-exchange and hydrogen bonding.	Adult (PO): Recommended starting dose is 800–1,600 mg 3 times daily.	Adverse events include nausea/vomiting, constipation, diarrhea, flatulence, and dyspnea. Sevelamer is a calcium- and aluminum-free phosphate binder so it may be advantageous when calcium-phosphate complexes are of concern.
			Pediatric (PO): Clinical data are lacking.	
**Hypocalcemia**	**Calcium supplementation**	Exogenous calcium (Ca++) replacement.	Adult: 500–2,000 mg elemental calcium PO in divided doses; 2–3 g Ca++ gluconate IV over 1 to 2 hours.	Reserved for patients who are symptomatic; elemental Ca++ content: carbonate (40%) > chloride (27%) > acetate (25%) > gluconate (9%); IV Ca++ gluconate is less irritating than other Ca++ salts.
			Pediatric: 200–2,000 mg elemental calcium PO in divided doses; Ca++ gluconate 100 mg/kg dose IV over 1 to 2 hours.	

ECG, electrocardiogram; IV, intravenous; PO, by mouth, mEq; milliequivalent.

## Abbreviations

AE: Adverse event; ALL: Acute lymphoblastic leukemia; CAD: Chronic actinic dermatitis; CMP: Complete metabolic panel; COPD: Chronic obstructive pulmonary disease; CRPC: Castrate-resistant prostate cancer; ECG: Electrocardiogram; FEC: Fluorouracil/epirubicin/cyclophosphamide; G6PD: Glucose-6-phosphate dehydrogenase; IV: Intravenous; LDH: Lactate dehydrogenase; MM: Metastatic melanoma; NHL: Non-Hodgkin’s lymphoma; NSCLC: Non-small cell lung cancer; TLS: Tumor lysis syndrome; UA: Uric acid; ULN: Upper limit of normal.

## Competing interests

The authors declare that they have no competing interests.

## Authors’ contributions

AM and PW were responsible for the conception and design of the manuscript. AM participated in drafting the manuscript, and both AM and PW were responsible for the review and/or revision of the manuscript. Both authors read and approved the final submitted manuscript.
